# A Logarithmic Quantization-Based Image Watermarking Using Information Entropy in the Wavelet Domain

**DOI:** 10.3390/e20120945

**Published:** 2018-12-08

**Authors:** Jinhua Liu, Shan Wu, Xinye Xu

**Affiliations:** School of Mathematics and Computer Science, ShangRao Normal University, Shangrao 334001, China

**Keywords:** image watermarking, entropy, logarithmic quantization index modulation, generalized Gaussian distribution, wavelet transform

## Abstract

Conventional quantization-based watermarking may be easily estimated by averaging on a set of watermarked signals via uniform quantization approach. Moreover, the conventional quantization-based method neglects the visual perceptual characteristics of the host signal; thus, the perceptible distortions would be introduced in some parts of host signal. In this paper, inspired by the Watson’s entropy masking model and logarithmic quantization index modulation (LQIM), a logarithmic quantization-based image watermarking method is developed by using the wavelet transform. Furthermore, the novel method improves the robustness of watermarking based on a logarithmic quantization strategy, which embeds the watermark data into the image blocks with high entropy value. The main significance of this work is that the trade-off between invisibility and robustness is simply addressed by using the logarithmic quantizaiton approach, which applies the entropy masking model and distortion-compensated scheme to develop a watermark embedding method. In this manner, the optimal quantization parameter obtained by minimizing the quantization distortion function effectively controls the watermark strength. In terms of watermark decoding, we model the wavelet coefficients of image by the generalized Gaussian distribution (GGD) and calculate the bit error probability of proposed method. Performance of the proposed method is analyzed and verified by simulation on real images. Experimental results demonstrate that the proposed method has the advantages of imperceptibility and strong robustness against attacks covering JPEG compression, additive white Gaussian noise (AWGN), Gaussian filtering, Salt&Peppers noise, scaling and rotation attack, etc.

## 1. Introduction

With the wide application of big data and other multimedia information technology, mass multimedia data are being generated and distributed over the Internet each day. This facilitates people’s daily work and life, but the security of these multimedia products are becoming more and more important, which has been studied over the past twenty years. One of the current effective methods is digital watermarking, which has been widely researched in the field of multimedia information security, such as data authentication, fingerprinting and broadcast monitoring, etc. [1,2,3]. Currently, most of the image watermarking algorithms focus on the study of imperceptibility and robustness.

Generally, the embedding method of watermarking can be divided into two categories due to the different embedding space used. The first type is based on the spatial domain and the other is dependent on the transform domain. For spatial domain-based watermarking, most algorithms mainly embed the watermark data by modifying the pixels of the image. While the transform domain-based watermarking is usually embedded by the coefficients of a properly transform domain, such as Fourier transform [4,5], discrete cosine transform [6,7] and wavelets [8,9,10,11]. According to different strategies of embedding watermark data, they can be classified into additive [12], multiplicative [13,14] and quantization-based methods [15,16,17,18,19]. Therefore, choosing the appropriate embedding space and strategy is very important for the design of watermarking algorithm.

In the current quantization-based watermarking algorithms, the most typical is the uniform quantization index modulation (UQIM) method, which has been presented in [16]. The UQIM method has far-reaching implications since it can achieve good distortion-robustness trade-off. Furthermore, UQIM is a blind watermarking scheme because the host image is not needed during the watermark detection. In this method, watermark information is embedded through quantizing the feature of host image by a set of quantizations, and each quantizer is associated with a different message. Although UQIM method is simple and easy to implement, it has a disadvantage is that it is sensitive to amplitude scaling attack. Moreover, the UQIM ignores the visual perceptual characteristics, which prone to introduce perceptible distortions in some parts of host signal. Several previously proposed works have addressed these problems. Literature [17] proposes a gain-invariant adaptive quantizer based on rational dither modulation (RDM) strategy in both watermark encoding and decoding. Experiments show that this method can well resist scaling attack. Literature [18] introduces an adaptive QIM (AQIM) watermarking method by utilizing modified Watson’s visual perceptual model, which exploits adaptive quantization step size to improve the fidelity of image and resistance to scaling attack.

To further improve the robustness of quantization-based watermarking, N.K.Kalantari et al. [19] introduces a logarithmic domain-based quantization index modulation (LQIM) watermarking which features perceptual advantages by μ-Law concept. They first transform the host signal into the logarithmic domain, then use the uniform quantization method to embed the watermark data, and extract the watermark data by applying the Euclidean distance decoder. The advantages of an LQIM method are desirable from perceptual perspective, where small quantization step sizes are devoted to smaller amplitudes and larger quantization step sizes are associated with larger amplitude. However, the visual perception model of the image itself is not considered, thus, some perceptible distortions may be introduced when decoding the watermark data. Recently, literature [20] proposes a gain invariant-based quantization watermarking method, which uses the division function strategy. This division function scheme has no effect on the watermark decoding process. Therefore, the watermarking method in [20] is invariant to gain attack, but the performance of watermarking against geometric attacks still needs to be further improved. Besides this, Carpentieri et al. [21] proposed a novel data-hiding method based on the modification of prediction errors (MPE) technique.They developed a new one-pass framework suitable for hyper-spectral images collected through remote sensing facilities. Experimental results demonstrated the effectiveness of their proposed method. Moreover, literature [22] presents a watermarking method by using a 4D hyperchaotic system with coherent superposition and modified equal modulus decomposition. Experiment simulations have validated that their proposed watermarking method has good robustness performance when against noise, occlusion and special attack. More importantly, their paper opens a new area of research as hybrid multi-resolution wavelet transform is used in their proposed method, where different combinations of transforms can be explored.

It is clear that the embedding space is important to watermark embedding as mentioned above. As reported in [4,5,6,7,8,9,10,11], most watermarking algorithms focus on the frequency domain due to its good tradeoff between robustness and invisibility. It is well known that wavelet-based watermarking methods have the advantages of multi-scale and multi-resolution characteristics. Therefore, we design the watermarking method in the wavelet transform domain.

We have proposed a preliminary version of parts of this work in [23]. There is a substantial difference between this paper and the conference version. The overall algorithm in conference [23] is much less elaborate. Motivated by the LQIM [19], we propose an improved logarithmic domain-based image watermarking in this paper. In order to obtain a good tradeoff between the invisibility and the robustness of watermarking, we embed the watermark data into the high entropy region of image in the logarithmic domain. For watermark detection, we model the wavelet coefficients of image by the generalized Gaussian distribution (GGD) model. Lastly, we evaluate and discuss the performance of the proposed watermarking through experiments.

Although the proposed method follows the framework of [19], there are a number of significant contributions that it presents. First, we embed strong watermark data into the complex texture region of image. Thus, the perceptual quality of the watermarked image can be kept at acceptable level. Second, although some analysis indicates that the embedding method between [19] and the proposed approach seems to be mathematically equivalent, there exist some differences between them. In paper [19], the uniform quantization method is used to quantize the transformed coefficients, while we use the distortion-compensated method to achieve the quantization in proposed method and an optimization strategy is applied for obtaining the optimal quantization step size. Quantization scalar factor is determined through the optimization method. In general, the proposed scheme is slightly more robust than [19] against some common distortions.

The rest of this paper is organized as follows. Section 2 introduces the proposed logarithmic quantization-based watermarking. Section 3 exploits the optimal value of quantization parameter. Experimental results about the imperceptibility and robustness of the proposed watermarking against common attacks are given in Section 4. Finally, we have some conclusions of this paper in Section 5.

## 2. Improved LQIM-Based Watermarking

**Figure 1:**
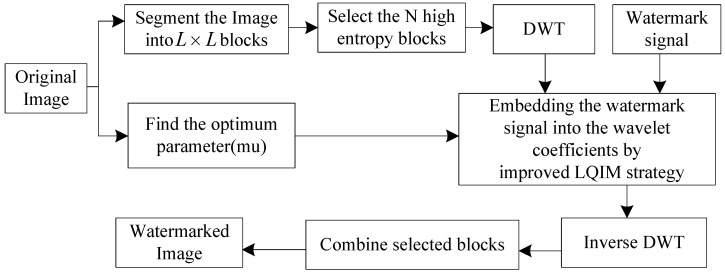
Flow chart of the proposed watermarking method.

It is well known that, usually, the rational exploitation of the human visual system will help to realize the invisibility of watermarking. Therefore, based on the entropy masking of visual perception model [24], we apply the high entropy blocks of an image to embed the watermark information. Figure 1 shows the flow chart of the proposed watermarking. Specifically, the proposed watermarking method performs as the following steps.

**Step 1:** Applying the Pseudo-random Noise (PN) generator to produce a binary watermark sequence. Let $b_{i}\in \left\{-1,1\right\}$ be the binary watermark signal.

**Step 2:** Divide the host image into non-overlapping L×L blocks, then sort these image blocks in descending by its entropy, and select frontal *k* high entropy image blocks as the embedding space. The selection threshold is set to the average entropy of all blocks. Generally, entropy can be computed by:

(1) $$H=-\sum_{i=1}^{n}p_{i}\log p_{i},$$

where $p_{i}$ denotes the probability of gray pixel *i* appearing in the image, and $\sum_{i=1}^{n}p_{i}=1$.

**Step 3:** Using the wavelet transform to decompose each selected image block. thus, the wavelet coefficients of mid-frequency sub-band image are obtained for embedding the watermark data.

**Step 4:** Let $\left[x_{1},x_{2},\ldots ,x_{\bar{N}}\right]$ be the set of selected wavelet coefficients of mid-frequency in each block. Then, we use logarithmic function to transform the set of wavelet coefficients $\left[x_{1},x_{2},\ldots ,x_{\bar{N}}\right]$ to

(2) $$c=\frac{\ln\left(1+\mu \frac{\left|x\right|}{X_{s}}\right)}{\ln\left(1+\mu \right)},\mu >0,X_{s}>0,$$

where parameter μ defines the compression level and $X_{s}$ is the parameter that scales the original signal. The calculation of parameter μ can be referred to in Section 3.1. Based on [19], the optimal value of $X_{s}$ can spreads most of the original image samples into the range [0,1]. As a result, let $c=[c_{1},c_{2},\ldots ,c_{\bar{N}}]$ be the set of transformed coefficients which can be obtained by Equation (2). Then, we adopt a distortion-compensated-quantization index modulation (DC-QIM) scheme to quantize the transformed signal $c_{i}$ for watermarking purpose. We have

(3) $$z_{i}=Q_{b_{i}}\left(c_{i}\right)+\left(1-\alpha \right)\left(c_{i}-Q_{b_{i}}\left(c_{i}\right)\right),i=1,2,\cdots \bar{N},$$

where $Q_{b_{i}}\left(c_{i}\right)=\mathrm{round}\left(\frac{c_{i}+b_{i}△}{△}\right)△-b_{i}△$ represents the adaptive quantizer, $b_{i}\in \left\{-1,1\right\}$ represents the binary watermark signal, Δ denotes the quantization step size; α represents the scalar factor of quantization, details are discussed in the Section 3.2, When α=1, the DC-QIM corresponds to the quantization index modulation scheme.

**Step 5:** Embed the watermark signal into the selected wavelet coefficients. We obtain the watermarked signal $y_{i}$ by:

(4) $$y_{i}=\mathrm{sgn}\left(z_{i}\right)\frac{X_{s}}{\mu }\left[{\left(1+\mu \right)}^{\left|z_{i}\right|}-1\right],$$

where sgn(·) represents the sign function, *z* is the quantized signal in the transformed domain, and $y_{i}$ represents the watermarked signal.

**Step 6:** Using the inverse wavelet transform to reconstruct the watermarked image block.

**Step 7:** Repeat Steps 3–6. Finally, combining the watermarked blocks with non-watermarked blocks to get the whole embedded watermark image.

For the extraction of the watermark, we use the Euclidean distance decoder in this work. Specifically, we adopt the proposed watermarking method to embed zero and one into the received signal *r* in the logarithmic domain, which results in $r_{0}$ and $r_{1}$, respectively. As a consequence, we extract the watermark signal as following:

(5) $$\hat{m}=\arg\min{∥r-r_{i}∥}^{2},i\in \left\{0,1\right\},$$

where $\hat{m}$ represents the extracted watermark signal.

## 3. Quantization Parameter Discussion and Error Probability Analysis

In this regard, we computed the optimum parameter μ and quantization scalar factor α, in which the optimum parameter for μ is found by minimizing the quantization distortion from reference [19]; the quantization scalar factor α is determined by the distortion-compensation interference and the noise interference. Besides this, the watermark error probability has been discussed in terms of the generalized Gaussian distribution (GGD) model in Section 3.3.

### 3.1. Optimal Parameter μ

In order to obtain the optimum value μ, we minimize the quantization distortion and the watermark power in this sub-section. We assume that the quantization noise *w* and $E[{∥x_{w}-x∥}^{2}]$ the watermark power areand in logarithmic transform domain, respectively. According to [19], $\left(x_{w}-x\right)$ can be written as:

(6) $$x_{w}-x=\frac{s_{q}}{s}x-x=\left(\frac{s_{q}}{s}-1\right)x,$$

where $s_{q}=\frac{X_{s}}{\mu }\left[{\left(1+\mu \right)}^{c+w}-1\right]$, *w* denotes the quantization noise. *c* denotes the quantized signal, $s=\sqrt{\frac{1}{N}\sum_{i=1}^{N}x_{i}^{2}}$ represents the normalized magnitude that embed one bit into the vector $X=\left\{x_{1},x_{2},\ldots ,x_{N}\right\}$. By adding and subtracting ${\left(1+\mu \right)}^{w}$ inside the bracket of expression $s_{q}$, we have

(7) $$\begin{array}{c} s_{q}=\frac{X_{s}}{\mu }\left[{\left(1+\mu \right)}^{c+w}+{\left(1+\mu \right)}^{w}-{\left(1+\mu \right)}^{w}-1\right]\\ =\frac{X_{s}}{\mu }\left[{\left(1+\mu \right)}^{w}\left({\left(1+\mu \right)}^{c}-1\right)+{\left(1+\mu \right)}^{w}-1\right]\\ =\frac{X_{s}}{\mu }\left[{\left(1+\mu \right)}^{w}-1\right]+\frac{X_{s}}{\mu }\left[{\left(1+\mu \right)}^{c}-1\right]{\left(1+\mu \right)}^{w}\\ =\frac{X_{s}}{\mu }\left[{\left(1+\mu \right)}^{w}-1\right]+s{\left(1+\mu \right)}^{w} \end{array}.$$

thus, $\left(x_{w}-x\right)$ can be further written as

(8) $$x_{w}-x=\left\{\frac{X_{s}}{\mu s}\left[{\left(1+\mu \right)}^{w}-1\right]+{\left(1+\mu \right)}^{w}-1\right\}x.$$

Simplifying the above equation, we have

(9) $$x_{w}-x=\left\{\left(1+\frac{X_{s}}{\mu s}\right)\left[{\left(1+\mu \right)}^{w}-1\right]\right\}x$$

According to Equation (9), replacing *s* with |x| when *s* is scalar, then we have

(10) $$x_{w}-x=\left(x+\mathrm{sgn}\left(x\right)\frac{X_{s}}{\mu }\right)\left[{\left(1+\mu \right)}^{w}-1\right],$$

where $\mathrm{sgn}\left(x\right)=x/\left|x\right|$, thus, $E[{∥x_{w}-x∥}^{2}]$ can be expressed as

(11) $$E\left[{∥x_{w}-x∥}^{2}\right]=E\left[{\left(x+\mathrm{sgn}\left(x\right)\frac{X_{s}}{\mu }\right)}^{2}\right]E\left[{\left({\left(1+\mu \right)}^{w}-1\right)}^{2}\right].$$

As well, we assume that the two terms in Equation (11) are independent for each other based on [19]. Therefore, the first term can be written as

(12) $$E\left[{\left(x+\mathrm{sgn}\left(x\right)\frac{X_{s}}{\mu }\right)}^{2}\right]=E\left[x^{2}\right]+2E\left[\left|x\right|\right]\frac{X_{s}}{\mu }+\frac{X_{s}^{2}}{\mu^{2}}.$$

Next, we calculate and minimize of watermark power by the method used in vector LQIM of [19]. Based on the obtained watermark power, Document to Watermark Ratio (DWR) can be computed as

(13) $$DWR=\frac{E\left[{∥x∥}^{2}\right]}{E\left[{∥x_{w}-x∥}^{2}\right]}={\left\{\left(1+2E\left[\left|x\right|\right]\frac{X_{s}}{E[x^{2}]\mu }+\frac{X_{s}^{2}}{E\left[x^{2}\right]\mu^{2}}\right)\times \left(\frac{1}{\Delta }\int_{-\Delta /2}^{\Delta /2}{\left({\left(1+\mu \right)}^{w}-1\right)}^{2}dw\right)\right\}}^{-1}.$$

Applying the Taylor series expansion for ${\left(1+\mu \right)}^{w}$, we can write it as

(14) $${\left(1+\mu \right)}^{w}=1+\ln\left(1+\mu \right)w+O\left(2\right),$$

where the higher order terms are neglected. Considering the above approximation, the expectation $E\left[{\left({\left(1+\mu \right)}^{w}-1\right)}^{2}\right]$ in Equation (11) can be rewritten as

(15) $$E\left[{\left({\left(1+\mu \right)}^{w}-1\right)}^{2}\right]=\ln^{2}\left(1+\mu \right)\frac{\Delta^{2}}{12}.$$

Using the above simplification form, and represent the optimum of LQIM by $\mu_{opt}$, it can be obtained by

(16) $$\mu_{opt}=\arg\min_{\mu \in (0,\infty )}\left\{\left(1+2E\left[\left|x\right|\right]\frac{X_{s}}{E[x^{2}]\mu }+\frac{X_{s}^{2}}{E\left[x^{2}\right]\mu^{2}}\right)\ln^{2}\left(1+\mu \right)\right\}.$$

### 3.2. Quantization Scalar Factor α

Firstly, we assumed that the received image contaminated by zero mean Additive white Gaussian noise (AWGN). The total interference energy is generated from both distortion-compensation interference and noise interference, and they are independent [16]. Thus, the interference function *f* is defined as:

(17) $$f=E\left({∥\varepsilon -(1-\alpha )(Q(x;m,\Delta /\alpha )-x)∥}^{2}\right),$$

where ε is Gaussian noise and $\varepsilon ∼N(0,{\sigma_{\varepsilon }}^{2})$, $\sigma_{\varepsilon }^{2}$ denotes the noise variance, then Equation (17) can be derived as:

(18) $$f=\sigma_{\varepsilon }^{2}+{\left(1-\alpha \right)}^{2}D/\alpha^{2},$$

where *D* is expectation distortion function, which is defined as $D=E\left(1/\bar{N}{∥y-x∥}^{2}\right)$ according to [16], where y denotes the watermarked signal, and x denotes the host signal, $E\left(\cdot \right)$ represents the mathematical expectation. One optimality criterion for choosing α is to maximize the “DIR (Distortion-Interference Ratio, DIR)”:

(19) $$\mathrm{DIR}\left(\lambda \right)=D_{1}^{2}/\left(\alpha^{2}\sigma_{\varepsilon }^{2}+{\left(1-\alpha \right)}^{2}D\right),$$

where *D* is the minimum distance. Let $\varphi \left(\alpha \right)=\lambda^{2}\sigma_{\varepsilon }^{2}+{\left(1-\alpha \right)}^{2}D$ and ∂φ(α)/∂α=0, and set the derivative of α to zero as follows:

(20) $$\partial \varphi \left(\alpha \right)/\partial \alpha =2\left(D+\sigma_{\varepsilon }^{2}\right)\alpha -2D=0.$$

Therefore the optimal α is obtained by

(21) $$\alpha_{opt}=D/\left(D+\sigma_{\varepsilon }^{2}\right)=1/\left(1+1/DNR\right),$$

where DNR is the distortion-to-noise ratio and have $DNR=\log_{10}\left(D/\sigma_{\varepsilon }^{2}\right)$.

### 3.3. Derivation of Error Probability

Roughly speaking, the distribution of the wavelet coefficients of image is highly non-Gaussian. Therefore, we utilize the generalized Gaussian distribution (GGD) [6,19] to model the image wavelet coefficients in this work. For simplicity, the host image modeled by the GGD, which is defined as

(22) $$p_{x}\left(x;\tilde{\mu },\alpha ,\beta \right)=\frac{\beta }{2\Gamma (1/\beta )\alpha }e^{-{\left|\frac{x-\tilde{\mu }}{\alpha }\right|}^{\beta }},$$

where $\tilde{\mu }$ denotes the mean value of the distribution.α represents the scale parameter and β denotes the shape parameter, Γ(·) is the Gamma function. When β=1, the GGD corresponds to a Laplacian distribution while β=2 corresponds to a Gaussian distribution.

**Figure 2:**
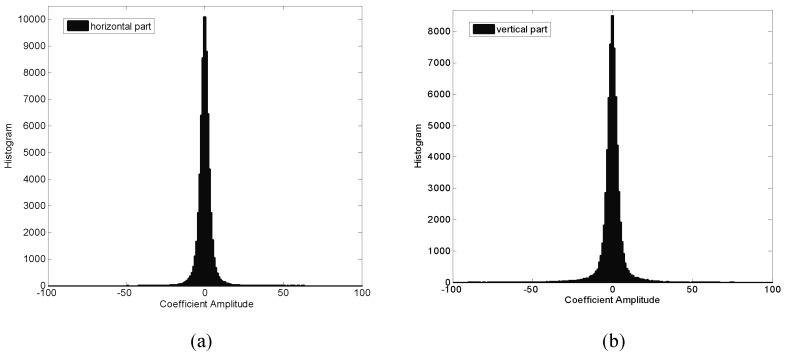
Histogram of horizontal part and vertical part of the image Lena. The kurtosis of the two distributions is measured at (**a**) 20.2610 (**b**) 25.9780, for the Gaussian distribution, the kurtosis is 3, and therefore the coefficients of wavelet transform are highly non-Gaussian.

**Figure 3:**
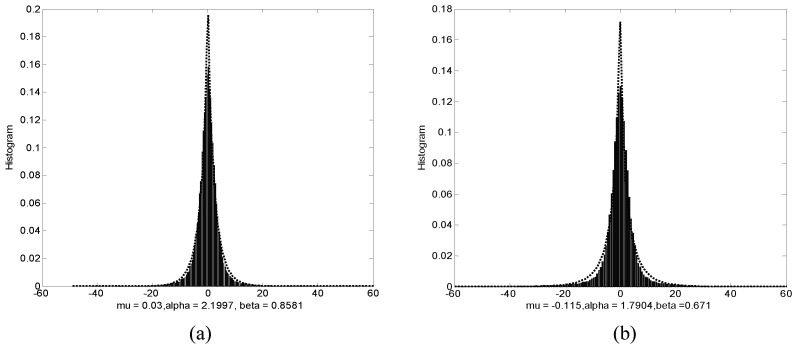
Wavelet sub-band coefficient histogram fitted with a generalized Gaussian distribution for Lena image, where mu represents the mean, alpha and beta represent the scale parameter and the shape parameter, respectively. (**a**) horizontal part (**b**) vertical part.

Figure 2 shows the histograms of wavelet coefficients, as can be seen, the wavelet coefficients are highly non-Gaussian. Moreover, Figure 3 shows the histogram of wavelet coefficients together with a plot of the fitted GGD. From Figure 3, we can see that the fits are quite good. Therefore, we can use the two parameters of GGD to model the wavelet coefficients. For the derivation of the error probability of watermark, we assume that the interference channel is AWGN. Error occurs in detection when noise causes the received signal to fall into a wrong region. From [19], the error probability of watermark is defined as

(23) $$p_{i}=\sum_{i=-\infty }^{\infty }o_{i}\sum_{m=-\infty }^{\infty }\int_{T_{i+2m}}^{T_{i+1+2m}}\frac{1}{\sqrt{2\pi }\sigma_{n}}e^{\frac{{\left(n-C_{i/2}\right)}^{2}}{2\sigma_{n}^{2}}}dn,$$

where $\sigma_{n}^{2}$ is the noise variance, $T_{i}$ is defined as

(24) $$T_{i}=\frac{C_{i/2}+C_{(i+1)/2}}{2},$$

where $o_{i}$ is the probability of occurrence of the host signal in the interval $\left[C_{(i-1)/2},C_{(i+1)/2}\right]$, assuming equal probabilities for −1 and 1 bits, it can be defined as

(25) $$o_{i}=\frac{1}{2}\int_{C_{(i-1)/2}}^{C_{(i+1)/2}}\frac{\beta }{2\alpha \Gamma \left(1/\beta \right)}\exp\left(-{\left|\frac{x}{\alpha }\right|}^{\beta }\right)dx,$$

where $C_{i}$ is defined as

(26) $$C_{i}=\mathrm{sgn}\left(i\right)\frac{X_{s}}{\mu }\left[{\left(1+\mu \right)}^{\left(\left|i\Delta \right|\right)}-1\right],$$

## 4. Experimental Results and Analysis

To evaluate the performance of the proposed watermarking method and validity of analytical derivations, the proposed watermarking algorithm is simulated on several benchmark images, which covers Lena, Barbara, Boat, Mandrill, Flintstones and Einstein. First, We first conduct the experiment by simulation on these images to show the imperceptibility of watermarking. Second, we have performed several robustness experiments to show the perceptual advantages of the proposed watermarking in comparison with previous quantization-based algorithms. Finally, to further verify the effectiveness of the detection performance of proposed method, the watermark error probability has been discussed under AWGN and JPEG compression attacks.

### 4.1. Imperceptibility Performance Test

In this section, we perform the imperceptibility performance test based on above six images. In terms of watermark embedding, the original images are segmented into non-overlapping blocks with size L×L firstly and *L* can be set to 8, 16, 32 or 64, respectively. The frontal *k* high entropy image blocks are chosen as the embedding space. For each selected image block, the 9–7 biorthogonal filters with three levels of decomposition are used to decompose the block, then two mid-frequency sub-band wavelet coefficients are quantized by using the logarithmic quantization strategy. The mid-frequency sub-band wavelet coefficients include horizontal direction decomposition coefficient and vertical direction decomposition coefficient. In this experiment, the size of image block is 32×32 and the number of image blocks is 64. Because the mid-frequency wavelet coefficients of the second level are used for quantizing, which result in embedding 8192 bits in a 512×512 original image.

**Figure 4:**
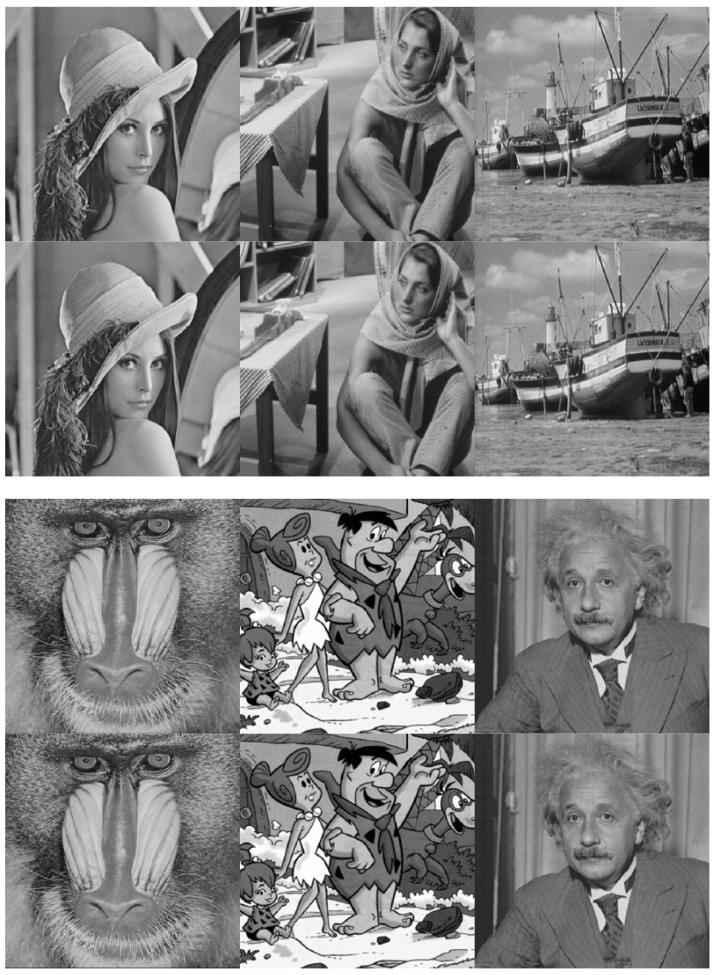
Original and watermarked images using the proposed method for Lena, Barbara, Boat, Mandrill, Flintstones and Einstein. For each image, the top one is the original image, the bottom one is the watermarked image.

**Figure 5:**
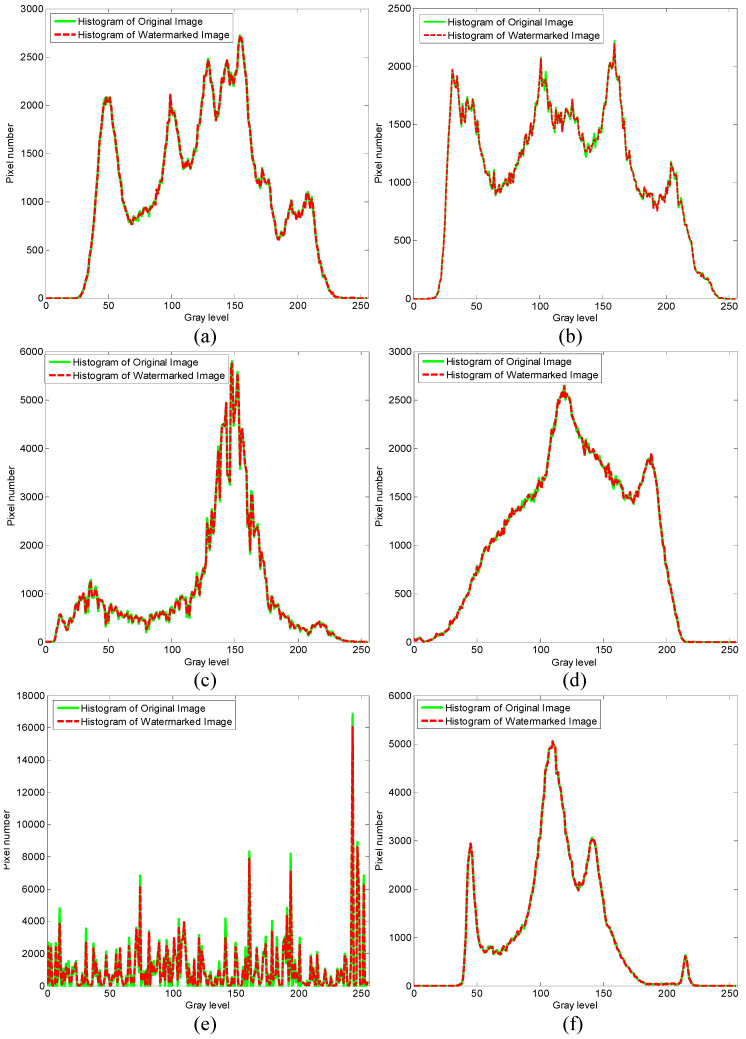
Histograms of the original image and the watermarked image. (**a**) Lena (**b**) Barbara (**c**) Boat (**d**) Mandrill (**e**) Flintstones (**f**) Einstein.

The results of the invisibility are shown in Figure 4. We can see that the watermark invisibility is satisfied. Moreover, to investigate the performance of proposed method in an objective way, we also evaluate the performance of the proposed watermarking method through the PSNR (Peak-Signal-to-Noise-Ratio, PSNR) and SSIM (Structure similarity index measure, SSIM) [25]. The results of PSNR and SSIM are shown in Table 1. It can be seen that the proposed method has good invisibility without any attack. Furthermore, we perform the histogram test to measure the difference between the host image and the watermarked image. As shown in Figure 5, it also can be found that the histogram of original image agrees closely with the histogram of watermarked image.

### 4.2. Robustness Performance Test

In oder to evaluate the robustness of the proposed method, some common image processing attacks and geometric distortion attacks are applied to the watermarked images, including AWGN, JPEG compression, scaling attack, median filtering and rotation attack. In this regard, the robustness of the proposed watermarking method under above attack is investigated using six well-known images including Lena, Barbara, Boat, Mandrill, Flintstones and Einstein. Besides, the size of all these images is 512×512. Lastly, we apply the BER (Bit error ratio, BER) to evaluate the watermark robustness under several intentional attacks.

Furthermore, to show the perceptual advantages of the proposed method, we compare it with previous quantization-based algorithms, we conduct experiments on benchmark test images. The comparisons of UQIM [16], AQIM [18], LQIM [19] and reference [20] under these attacks are performed. All watermarking methods contain the same watermark length and PSNR value. The watermark length is 8192 and the PSNR for all images is 45 dB. Quantization step size is selected as 0.07, 3.0, 1.50, 0.65 and 0.09 for the proposed method, UQIM [16], AQIM [18], LQIM [19] and reference [20], respectively. Furthermore, we set $X_{s}=200$, and $\mu_{opt}=7.52$, and the quantization scalar factor is 0.75 for the proposed method.

Table 2 shows the results of comparison of UQIM [16], AQIM [18], LQIM [19] and reference [20] under AWGN, median filtering, JPEG compression, amplitude scaling and rotation attack. From Table 2, we can see that the proposed method outperforms UQIM, AQIM, LQIM and reference [20]. The main reasons are as follows. In these quantization-based watermarking, the UQIM [16] uses a uniform quantizer for watermark embedding, which may reduce the fidelity of the host image. AQIM [18] utilizes perceptual model to exploit an adaptive quantizer, which improves the robustness of watermarking. However, the discrepancies between the corresponding estimated quantization step sizes at the embedder and the decoder, resulting in reducing the robustness of AQIM [18]. Considering the Watson’s entropy masking and the optimal quantization parameter, the proposed method has a slightly larger BER than LQIM [19] and reference [20].

**Figure 6:**
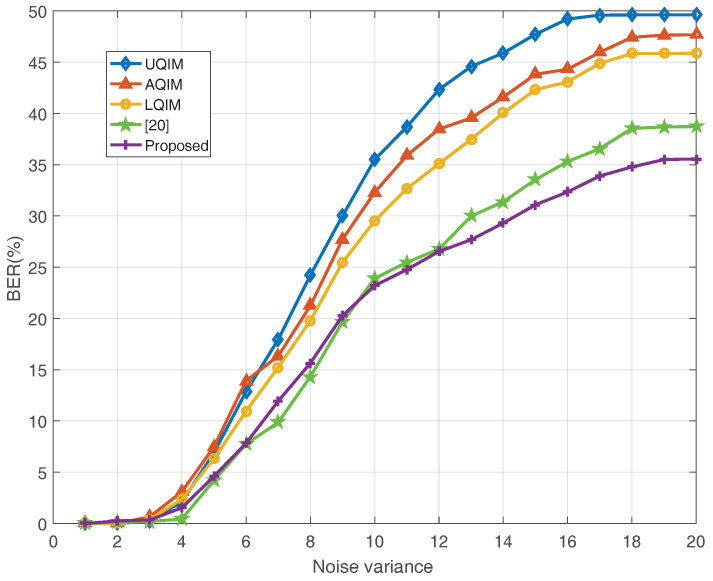
BER (%) of watermark extraction under AWGN attack for various noise variances. The results are averaged over six well-known images. 4096 bits have been embedded in each image in all methods.

**Figure 7:**
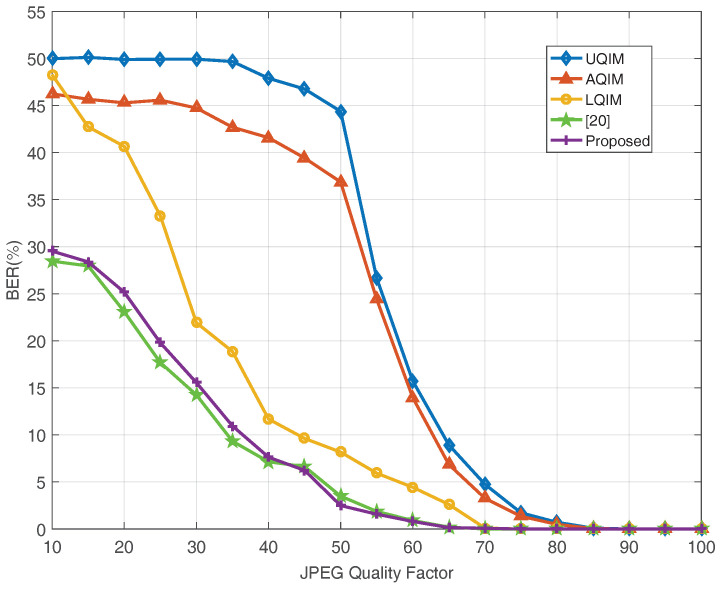
BER (%) versus JPEG quality factor for JPEG compression attack. The results are averaged over six well-known images; 4096 bits have been embedded in each image in all methods.

**Figure 8:**
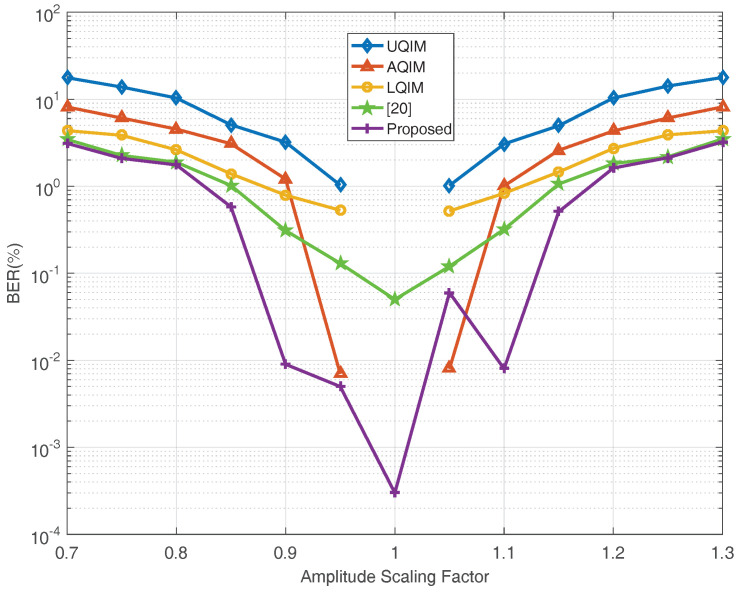
BER (%) versus scaling factor for scaling attack. The results are averaged over six well-known images.4096 bits have been embedded in each image in all methods. Note that for UQIM, AQIM and LQIM, the BER of these methods is 0 when scaling factor is equal to 1.0, thus, these points are therefore not plotted.

**Figure 9:**
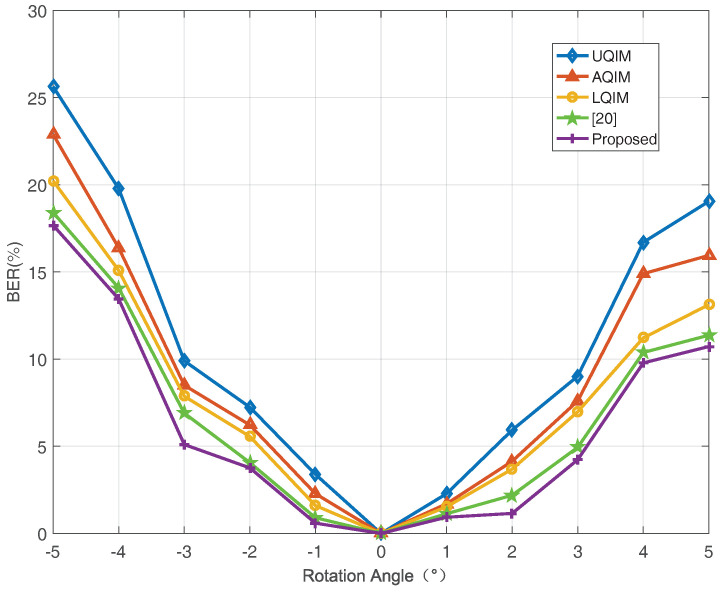
BER (%) versus different angle for rotation attack. The results are averaged over six well-known images.4096 bits have been embedded in each image in all methods.

To further verify the performance of watermark under AWGN, JPEG compression, amplitude scaling and rotation attacks. Simulations are also performed on six benchmark test images and the average results are depicted in Figure 6, Figure 7, Figure 8 and Figure 9. In this regard, the optimal quantization scalar factor is set to 0.75, and the image block size is 32×32. Different from Table 2, the watermark length is 4096 in all Figure 6, Figure 7, Figure 8 and Figure 9. It is to say that we choose 32 image blocks with high entropy value as the watermark embedding space, then the two set of mid-frequency wavelet coefficients of the second level are quantized for watermark embedding, resulting in embedding 4096 bits in an image. Besides, the other pre-defined parameters are chosen as mentioned above. The results are averaged over above six images.

Figure 6 shows the results of the proposed method in comparison with UQIM [16], AQIM [18], LQIM [19] and reference [20] under AWGN attack. From the simulation results in Figure 6, the proposed approach outperforms these previous quantization-based methods. Among many watermarking applications presented so far, JPEG compression is the most common image distortion attack.

Therefore, we also simulate all methods against JPEG compression attack in Figure 7. As seen, the overall performance of the proposed algorithm is satisfied. However, the performance of the proposed method is slightly worse than [20] under strong JPEG compression strength, and this issue will be investigated in our future work. Figure 8 and Figure 9 illustrate the robustness of the proposed method in comparison with the above quantization-based watermarking under amplitude scaling attack and rotation attack, respectively. It can be seen from these two figures that the performance of proposed watermarking in different scaling factor and angles is better than the above watermarking methods.

Besides this, the computational time of the proposed watermarking method with different images is presented in Table 3, and we compare it with previous quantization-based algorithms in terms of the computational time. Note that all the results are implemented in MATLAB R2016a. As shown in Table 3, the proposed watermarking algorithm has high computational efficiency.

In summary, the robustness of the proposed method outperforms the other comparison methods. The main factors are summarized as follows. First, the high entropy image region is selected as the watermark embedding space, which will improve the invisibility of the watermarking. Moreover, the optimal quantization scalar factor is used to control the perceptual distortion of watermark embedding, which reduces the effect of embedding distortion on the watermarked image. On the other hand, we exploit the the logarithmic quantization strategy in designing the watermarking, which improves the robustness of the proposed watermarking.

### 4.3. Discussion of Error Probability

Under AWGN and JPEG compression attacks for Lena image, respectively. The error probability is derived according to the method described in Section 3.3, and it is calculated by $P_{e}=\frac{1}{k}\sum_{i=1}^{k}p_{i}$, where *k* is the total number of the selected high entropy blocks in the watermarking system. $p_{i}$ is computed by applying the Equation (23) described in Section 3.3.

**Figure 10:**
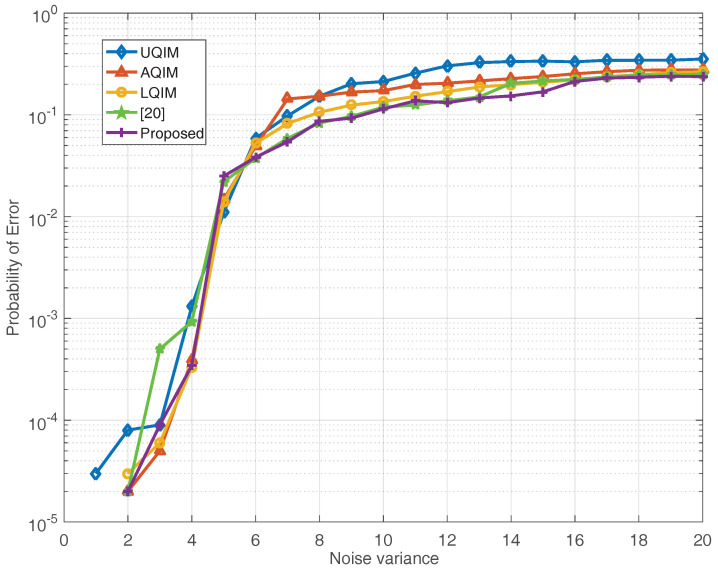
Probability of error under AWGN attack with different noise variance.

**Figure 11:**
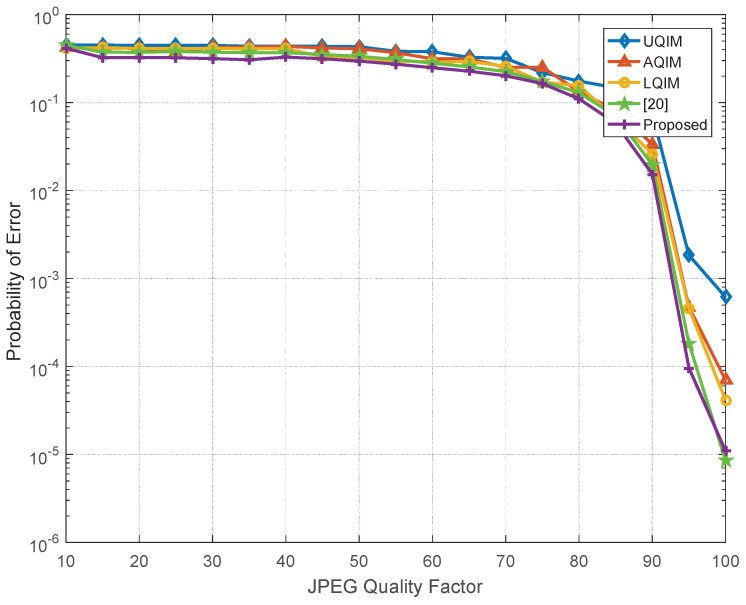
Probability of error under JPEG compression attack with different quality factor.

From Figure 10 and Figure 11, it can be seen that the proposed method has slightly better performance than UQIM [16], AQIM [18], LQIM [19] and reference [20]. This further validates the effectiveness of the proposed algorithm.

Meanwhile, it also shows that the GGD model can well describe the non-Gaussian property of wavelet coefficients. In brief, the main reasons are summarized as follows. First of all, the watermark signal is embedded into the high entropy region of the host image. By using this strategy, the imperceptibility of the watermarking system can be improved effectively. Second, we use the distortion-compensated approach to achieve the quantization-based watermark embedding in proposed method and an optimization strategy is applied for obtaining the optimal quantization step size. By applying this method, the quantization distortion of the host signal can be reduced and the robustness of watermarking can be improved. Finally, thanks to the good fitting ability of the generalized Gaussian distribution model for non-Gaussian property of wavelet coefficients, the detection performance of the watermarking can be enhanced effectively.

As reported in Table 2 and in figures ranging from Figure 6, Figure 7, Figure 8, Figure 9, Figure 10 and Figure 11, the robustness of the proposed watermarking method outperforms the other quantization-based watermarking methods mentioned above. The main factors are summarized as follows.First, the high entropy image region is selected as the watermark embedding space, which will improve the invisibility of the watermarking. Moreover, the optimal quantization scalar factor is used to control the perceptual distortion of watermark embedding, which reduces the effect of embedding distortion on the watermarked image. Second, we exploit the the logarithmic quantization strategy in designing the watermarking, and find the optimum parameter by minimizing the quantization distortion, which improves the robustness of the proposed watermarking. Finally, we utilize the generalized Gaussian distribution model to model the wavelet coefficients, which improves the detection performance of the proposed watermarking.

However, the performance of the proposed method is slightly worse than [20] under strong JPEG compression strength. Furthermore, the proposed watermarking method performs weakly when against some geometric distortion attacks, which covers complex affine transformation, cropping attack, synchronous attack and local random bending attack, and so on. To address these difficult problems, we will adopt some advanced methods and techniques in our future work, including group component analysis [26], sparse Bayesian learning [27,28,29], and deep convolutional neural networks [30], etc.

## 5. Conclusions

Wavelet transform has been successfully applied in many image processing areas, such as digital watermarking, JPEG compression and image restoration, etc. In this work, we develop a modified logarithmic quantization-based watermarking method based on information entropy in the wavelet domain. By using the information entropy, the invisibility of watermark can be improved effectively. Furthermore, an optimization strategy is applied for obtaining the optimal quantization step size. The robustness of the proposed watermarking is satisfied through a series of experimental results. In terms of watermark decoding, we apply the generalized Gaussian distribution model to describe the distribution of the wavelet coefficients. Simulation results show the effectiveness of the watermark detection. Future work will probably include investigating a novel data-hiding algorithm by applying other technologies such as sparse representation, deep learning and convolution neural network, etc.

## Figures and Tables

**Table 1:** Performance evaluation results with block size of 32×32.

Image	Block Size 32, Watermark Length 4096		Block Size 64, Watermark Length 8192	
	PSNR (dB)	SSIM	PSNR (dB)	SSIM
Lena	49.4738	0.9991	45.4725	0.9873
Barbara	50.1853	0.9986	46.1507	0.9882
Boat	49.9362	0.9994	45.7279	0.9891
Mandrill	49.0115	0.9978	46.5381	0.9876
Flintstones	49.8023	0.9979	46.6269	0.9931
Einstein	50.1209	0.9998	46.7546	0.9970

**Table 2:** BER (%) results of extracted watermark under common attacks.

Image	Method	Noise var. 10	Med. 3×3	JPEG 30%	Scal. 0.75	Rot. $10^{\circ }$
Lena	UQIM	41.2045	15.6564	50.0913	24.4673	35.3694
	AQIM	36.6534	13.2963	45.1478	7.5864	31.4782
	LQIM	35.1021	11.5905	23.356	4.1789	27.0649
	[20]	29.4536	3.9442	14.2872	2.0793	29.9434
	Proposed	26.5327	3.0067	15.3684	1.9032	22.1481
Barbara	UQIM	40.7069	16.2378	49.7886	24.8723	37.0023
	AQIM	37.7734	13.8965	45.3522	8.5662	32.129
	LQIM	34.6308	10.9732	24.1125	4.3215	26.5674
	[20]	29.1096	2.6455	12.5789	2.3459	30.1073
	Proposed	25.4373	2.8529	14.0924	3.1145	25.0805
Boat	UQIM	42.0832	14.7317	50.3342	23.5439	36.5547
	AQIM	36.1566	12.7064	46.0105	6.1148	32.1982
	LQIM	34.8204	10.4285	24.3584	3.0934	29.0478
	[20]	28.1759	2.4936	13.9037	1.0978	29.478
	Proposed	26.9086	2.5298	14.8045	1.3086	23.8009
Mandrill	UQIM	41.7883	16.0907	49.2336	23.576	35.9014
	AQIM	35.2824	13.214	44.7608	7.9004	31.5773
	LQIM	33.1708	10.2786	23.8714	4.8232	24.1335
	[20]	28.5065	2.9115	13.0139	3.1175	25.1052
	Proposed	25.0421	1.8973	15.217	2.804	23.4468
Flintstones	UQIM	41.1378	15.4687	50.0127	23.757	36.8089
	AQIM	37.4346	13.3658	45.6648	7.1624	32.3427
	LQIM	34.0708	11.7741	23.787	3.3931	27.2319
	[20]	30.0012	3.0539	14.1002	2.0085	28.1004
	Proposed	26.2431	2.6963	16.9593	1.7016	24.4546
Einstein	UQIM	40.4682	14.9035	50.2443	23.9342	36.4002
	AQIM	36.1015	13.2006	44.9782	7.0743	31.6768
	LQIM	33.8854	10.6433	24.4105	3.5503	27.5649
	[20]	30.7238	3.214	13.5108	2.843	28.2404
	Proposed	27.1107	2.7436	15.8173	1.9061	25.2115

**Table 3:** Computational time of several watermarking methods with different image (unit: s).

Image	UQIM	AQIM	LQIM	[20]	Proposed
Lena	2.9095	3.5426	3.4029	3.0930	2.7687
Barbara	2.8317	3.4185	3.2598	3.2521	2.8124
Boat	2.7848	3.3021	3.3116	3.1942	2.9236
Mandrill	2.8560	3.1435	3.2079	2.8964	2.6411
Flintstones	2.9084	3.2028	3.3018	3.2026	2.8455
Einstein	2.8867	3.1329	3.2465	3.1708	2.6903
**Average**	**2.8628**	**3.2904**	**3.2884**	**3.1348**	**2.7802**
